# Wetting Characteristics of Laser-Ablated Hierarchical Textures Replicated by Micro Injection Molding

**DOI:** 10.3390/mi14040863

**Published:** 2023-04-16

**Authors:** Peng Gao, Ian MacKay, Andrea Gruber, Joshua Krantz, Leonardo Piccolo, Giovanni Lucchetta, Riccardo Pelaccia, Leonardo Orazi, Davide Masato

**Affiliations:** 1 Plastics Engineering Department, University of Massachusetts Lowell, Lowell, MA 01854, USA; peng_gao@uml.edu (P.G.);; 2 Department of Industrial Engineering, University of Padova, 35100 Padova, Italy; 3 Department of Sciences and Methods for Engineering, University of Modena and Reggio Emilia, 41124 Reggio Emilia, Italy; 4 EN&TECH, University of Modena and Reggio Emilia, 41124 Reggio Emilia, Italy

**Keywords:** wetting, hierarchical texturing, micro injection molding, femtosecond laser ablation

## Abstract

Texturing can be used to functionalize the surface of plastic parts and, in particular, to modify the interaction with fluids. Wetting functionalization can be used for microfluidics, medical devices, scaffolds, and more. In this research, hierarchical textures were generated on steel mold inserts using femtosecond laser ablation to transfer on plastic parts surface via injection molding. Different textures were designed to study the effects of various hierarchical geometries on the wetting behavior. The textures are designed to create wetting functionalization while avoiding high aspect ratio features, which are complex to replicate and difficult to manufacture at scale. Nano-scale ripples were generated over the micro-scale texture by creating laser-induced periodic surface structures. The textured molds were then replicated by micro-injection molding using polypropylene and poly(methyl methacrylate). The static wetting behavior was investigated on steel inserts and molded parts and compared to the theoretical values obtained from the Cassie–Baxter and Wenzel models. The experimental results showed correlations between texture design, injection molding replication, and wetting properties. The wetting behavior on the polypropylene parts followed the Cassie–Baxter model, while for PMMA, a composite wetting state of Cassie–Baxter and Wenzel was observed.

## 1. Introduction

Polymers have been widely used as the material for micro-components and products containing micro-features because of their ease of processing and cost-effectiveness [1]. Applications can be found in various fields, such as packaging, biomedical, micro-electronics, and thin-film technologies [2]. The modification and creation of functional plastic surfaces have been the topic of research and industrial production over the last 20 years. Wettability is one of the most important properties of solid surfaces [3,4,5]. The contact angle has been commonly used to represent surface wettability. A hydrophobic surface is a surface showing a contact angle larger than 90°. Conversely, the surface is hydrophilic when the contact angle is less than 90°. Research shows that surfaces containing hierarchical structures or multiple levels of features result in higher wetting angles than surfaces containing only micro-scale or nano-scale features [6,7,8]. Hydrophobic functionality is essential in many existing technologies, as well as new and emerging technologies, including self-cleaning surfaces, microfluidics, biomechanics, and antifouling applications. 

Based on the conventional injection molding technique, micro-injection molding has been demonstrated as a key enabling technology for mass-producing polymer textured surfaces [9]. It has been shown that the stability and effectiveness of surface feature replication depend on the polymer properties and processing conditions [10]. Several polymers have been used to study the replication of micro- and nano-scale textures via micro-injection molding [1,11]. Considering the high viscosity of polymer melts, high injection velocity and packing pressure are recommended to achieve larger replication [12,13]. The most common polymers used in micro-injection molding are reported in Table 1. The properties of the chosen polymer, such as its rheological, thermal, and shrinkage properties, affect the replication. Investigations have reported a series of replication and melt viscosity measurements within micro-scale geometry using amorphous and semi-crystalline polymers [14,15]. 

As discussed by Masato et al. [16], when designing plastic products with textured surfaces, it is essential to consider the selection of the most appropriate technology for the fabrication of the micro- and nano-scale features. Various fabrication techniques have been developed for the texturing of mold inserts, such as micromachining [17], chemical etching [18], lithography [19], coatings [20], and laser ablation [21]. An overview of these different texturing technologies is presented in Table 2, where the main chemical, mechanical, additive [22,23], and thermoelectric processes and the most common techniques for each process are described in [16].

Ultrashort femtosecond lasers have recently been identified as a flexible and cost-effective technology for texturing. Femtosecond laser sources can be integrated into CNC machines, allowing for the ablation of complex mold surfaces [30]. Moreover, controlling the laser texturing parameters allows for generating micro- and nano-scale features with the same equipment in a one-step process [31,32]. This allows for significant advantages over other technologies, such as those that rely on masks (i.e., limits on the feature size) or those requiring a clean room environment (e.g., EBM). In addition, compared to etching technologies, femtosecond laser does not require chemicals, making it an environmentally friendly technology. Overall, the combined texture geometry flexibility, texturing speeds, and ease of implementation make laser ablation an ideal technology for micro-injection molding applications.

This work aims to manufacture plastic parts with controlled wetting properties by exploiting surface texturing. To achieve this goal, hierarchical textures were generated on steel mold inserts using femtosecond laser ablation. Different micro-scale textures were designed to study the effects on the wettability of injection-molded plastic parts. Nano-scale ripples were then developed over the micro-scale texture by exploiting laser-induced periodic surface structures. The textures were replicated by micro injection molding using polypropylene and poly(methyl methacrylate). The static wetting behavior was investigated on both steel inserts and molded parts. The theoretical contact angle values were compared to the experimental data to determine the effects of the texture design and the polymer properties. 

## 2. Materials and Methods

### 2.1. Laser Texturing Techniques

The mold inserts used in this work were designed to have different textures characterized by different geometry. To achieve static and dynamic wetting functionalization, all textures were hierarchical, i.e., had a nano-scale texture over a micro-scale texture. The micro-scale texture patterns were selected to test various geometries, and the nano-scale texture was a uniform repetition of ripples. The micro- and nano-scale texturing was obtained using a femtosecond laser. At first, direct laser ablation was used to generate the micro-scale features; then, the ultrashort pulsed laser was used to create laser-induced periodic surface structures (LIPSS). The hierarchical texture designs were selected to achieve wetting functionalization while keeping a low aspect ratio. Indeed, when designing for injection molding replication, it should be considered that replicating high aspect ratio structures requires a high mold temperature. While the replication of high aspect ratio structures has been reported in the literature [33,34,35,36], it is achieved at the expense of an extended cycle time and the need for additional molding equipment. 

The surface textures were generated by exploiting an Exspla Atlantic IR5-GR2-UV1 laser source, operating the first harmonic at 1064 nm and the second at 532 nm, delivering pulses with a 10 ps pulse duration. The linear light polarization direction was controlled using a λ/2 waveplate. The laser light was deflected using two Raylase Superscan IV scanners with an input diameter of 14 mm. The beamlines were focused onto the steel surface using an 80 mm focal length lens for IR irradiation and a 77 mm focal length lens for GR irradiation. Considering the beam quality and the real characteristics of the F-theta lenses, the diameters of the focused spot were about 12 µm for IR and 10 µm for GR. In Table 3 the most critical parameters of the laser system are summarized.

The inserts were laser textured using a two-step approach. First, the microgeometry was engraved using GR irradiation. Second, LIPSS were formed on the engraved surfaces using IR irradiation for inserts A, B, and D and GR irradiation for insert C. The insert A microstructure was engraved using a cross-hatch approach with two scanning lines for each trench. The pattern lines had a pitch of 60 µm and a width of about 15 µm. The insert B microstructure was engraved by drilling a pattern of microholes with a base diameter of about 10 µm and a pitch of 30 µm. Inserts C and D were microstructured with parallel lines of partially overlapping micro holes. The lines had a pitch of 20 µm and a width of about 8 µm.

For the investigation, four textured inserts are shown in Table 4: a grid pattern (Insert A), a hole pattern (Insert B), and two different parallel trench patterns (Inserts C and D) were used. SEM images show the hierarchical topography of each insert by detailing the micro-scale features on the low magnification micrographs and the nano-scale LIPSS on the high magnification ones. The micrographs showed the regular repetition of the micro- and nano-scale designs across the mold insert surface. Images at a higher resolution showed the presence of regular LIPSS over imposed on the top surfaces of the samples.

### 2.2. Polymer Selections

An amorphous polymethyl methacrylate (PMMA, Altuglas US UVT, Philadelphia, PA, USA) and a semi-crystalline polypropylene (PP, Borealis, 125BBMO) were used to replicate the textured mold inserts. The main properties of the materials are presented in Table 5. 

### 2.3. Injection Molding Process Setup

**Figure 1:**
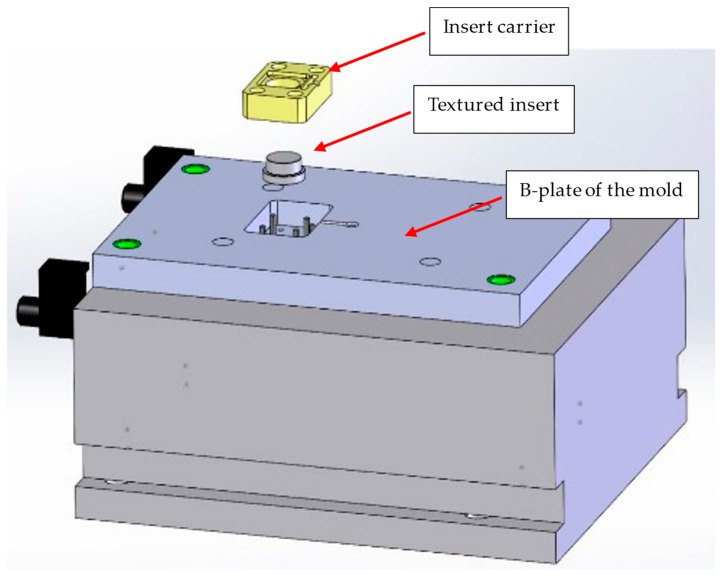
B-plate and assembly of the textured insert in the mold.

The injection molding experiments were carried out on a BOY injection molding machine (BOY22A, Exton, PA, USA). The mold temperature was controlled using water (Thermolator, Conair, Queens, NY, USA). Circular textured steel inserts were mounted using a rectangular insert carrier in the B-plate of the mold (Figure 1).

**Figure 2:**
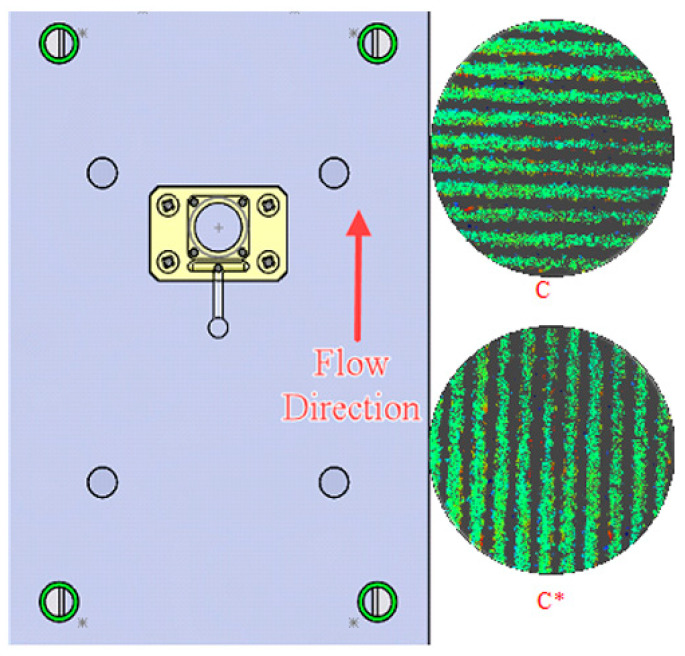
Different mold insert orientation and flow direction of the polymer melt. C indicates the orientation of the features is parallel to the flow direction, and C* indicates the orientation is vertical to the flow direction.

As inserts C and D features were long straight trenches, two orientations were introduced for these two inserts. Samples molded when the features were oriented perpendicular to the polymer flow direction were labeled C or D, while samples molded when the features were oriented parallel to the flow direction were labeled C* or D*. A blank steel insert made from the same grade of steel was used to create plastic samples with no features, which were used as a baseline. Figure 2 demonstrates the different orientations of insert C and D on the B-side of the mold. 

Considering the extensive literature on the topic, the analysis of the effects of different molding parameters on the replication was not considered. Processing conditions were defined from material datasheets, literature recommendations, and preliminary experiments. In particular, the mold temperature was kept high, but below the transition temperature. Indeed, while it is well known that a higher mold temperature is required to achieve higher replication, the process setup required rapid heat cycle molding technologies and an extended cycle time [10]. In this work, we purposefully designed low aspect ratio structures to facilitate replication, while still allowing for functionalization of the surface of the plastic parts. The packing pressure and time were determined using the short shot method and gate freeze study. Key processing parameters are presented in Table 6. After 20 defect-free samples were fabricated in automatic mode for each insert, the following 10 samples were collected. 

### 2.4. Texture Characterization

#### 2.4.1. Characterization of Texture Geometry 

A Veeco WYKO NT2000 optical profiling system was used to obtain the depth of the micro-scale features on the steel inserts and the height of the replicated features on the molded plastic parts. The scanning length and back-scan were set to 15 µm, meaning there was a total z-direction scanning length of 30 µm in order to ensure the entire surface topography was fully scanned. The stage was leveled before and after each sample was measured. Once the sample was brought into focus at a magnification of ~20×, the stage’s height and tilt were adjusted, such that the fringe was located on the topmost surface of the sample, indicating that the scan was zeroed on the top of the sample. The scanning and back scan lengths moved 15 µm below and above this level to detect features at other levels. The brightness of the light source was adjusted for each sample, depending on its reflectivity. The height and depth of the features were obtained directly from profiles evaluated on the acquired topographies. For each scan, multiple profiles were evaluated along the direction of the micro-scale features to measure their depth. The measurements on the steel inserts were repeated three times at different locations. In contrast, the measurements on the molded polymer parts were repeated on three different samples and three areas on each sample, with nine measurements on each plastic piece. 

**Figure 3:**
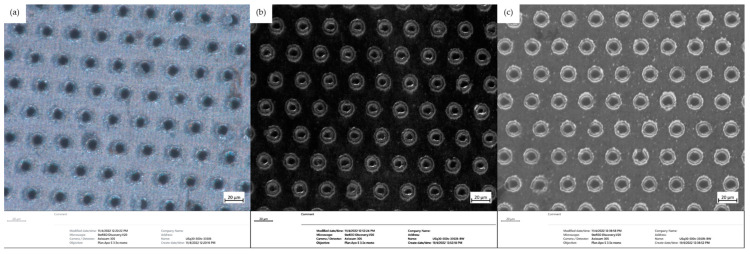
Stereo microscope images (500×) (**a**) textured insert B, (**b**) PP part molded with insert B, and (**c**) PMMA part molded with insert B.

The texture width and pitch distance were analyzed using a stereo microscope system (V20 SteREO Discovery, ZEISS, Oberkochen, Germany) and the ZEN Core (ZEISS, Germany) image processing software. A light source with a color temperature of 3350 K was used for all of the images, providing light from the top of the textured surfaces. In the cases of transparent samples, a black background was provided at the bottom of the surface to provide sufficient resolution. A magnification of 500× was used for all of the samples, including steel inserts and plastic molded parts. Figure 3 shows the images obtained from the stereo microscope of (a) the steel insert, (b) sample molded with PP, and (c) sample molded with PMMA. The dimensional properties of the textured surfaces were directly measured using the image processing software. 

**Figure 4:**
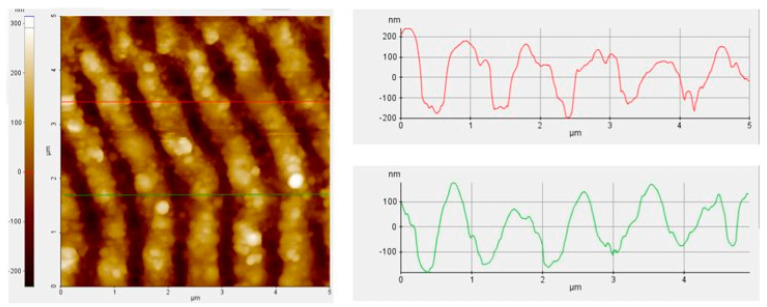
AFM image of nano-scale features on insert A.

An atomic force microscope (AFM) (PARK XE-100, Park Systems, Suwon, Republic of Korea, 16229) was used to capture and analyze the nano-features on the steel inserts. The AFM scan on insert A is demonstrated in Figure 4. From each AFM scan, the LIPSS dimensions were evaluated from multiple profiles, which were extracted perpendicular to the ripples. The AFM analysis indicated that the LIPSS features on all four inserts had similar dimensions with an average height of 310 ± 80 nm. For the purpose of this study (i.e., analysis of the effect of different micro-scale texture design on wetting functionalization), all LIPSS were considered equal even, although the standard deviation suggests a different formation over the different micro-scale textures. A more in-depth AFM analysis, would be required to characterize the LIPSS in detail and to better understand the nano-scale ablation over different micro-scale geometries.

#### 2.4.2. Characterization of Wetting Properties 

Static and dynamic contact angle measurements were performed on a drop shape analyzer (DSA100, Kruss Inc., Hamburg, Germany, 22453). The static contact angle measurements were performed using a drop volume of 2 μL. The drop was deposited on the micro-structured surface and stabilized for 15 s before 15 measurements were taken at 1 s intervals. The measurements were repeated three times on each sample without disrupting the sample’s position or the platform height, so as to ensure consistency. The measurements were conducted in parallel and orthogonal directions for textures with trenches-like features [4].

## 3. Results and Discussions

### 3.1. Characterization of Steel Inserts

Each steel insert’s aspect ratio (AR) and top surface area (TSA) were calculated based on the measurement obtained from the optical profilometer and stereo microscope. The aspect ratio is the ratio between the depth of features, and the average width or diameter of textured features are cylindrical holes. The top surface area was calculated from images obtained using a stereo microscope. A 150 μm × 150 μm area was selected on each image, and the top surface was calculated based on the diameters of the individual features on the top surfaces. The channel features on inserts A, C, and D were approximated as cuboids, while the holes in insert B were approximated as cylinders. The average (Avg.) and standard deviation (Std. Dev.) of the geometrical properties of the steel inserts are presented in Table 7. 

The wetting properties of all four inserts are presented in Table 8. The wetting properties of a blank steel insert are also reported as a baseline. The percent change values are determined by Equation (1).

(1) $$\mathrm{Percent}\;\mathrm{Change}=\frac{\mathrm{Static}\;\mathrm{Contact}\;\mathrm{Angle}\;\mathrm{of}\;\mathrm{Insert}-\mathrm{Static}\;\mathrm{Contact}\;\mathrm{Angle}\;\mathrm{of}\;\mathrm{Blank}}{\mathrm{Static}\;\mathrm{Contact}\;\mathrm{Angle}\;\mathrm{of}\;\mathrm{Blank}}\;\times 100\%\;$$

The static contact angle (CA) was <90° on the blank steel insert, indicating the surface was hydrophilic. All of the textured surfaces showed static contact angles >90° and thus were considered hydrophobic. This transition from hydrophilic to hydrophobic surfaces indicated that the wetting model on the characterized steel inserts followed the Cassie–Baxter wetting model. In the Cassie–Baxter model, air trapped in the grooves formed a composite (solid/air) surface, usually resulting in a larger contact angle than an ideally smooth surface. The relationship of the static contact angle of a textured surface ($\theta_{C-B\;}$) and a perfectly smooth one (θ) followed the Cassie–Baxter wetting model [37,38]:

(2) $$\cos\theta_{C-B\;}=Fs\cdot \cos\theta +Fs-1$$

where *Fs* represents the fraction of solid/liquid contact in the composite surface beneath the liquid. The *Fs* value for each insert can be defined as the ratio of the top surface area (TSA) and the 150 μm × 150 μm area of interest. 

**Figure 5:**
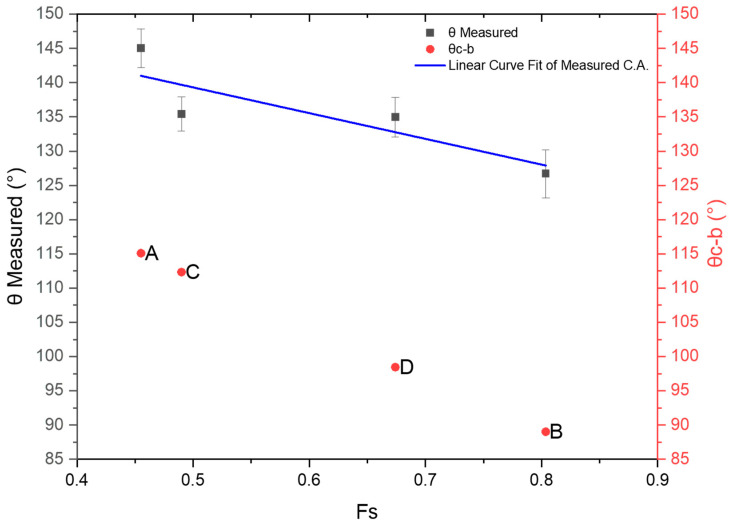
Wetting properties of steel inserts compared with the Cassie–Baxter model, A, B, C and D indicates the Insert index.

Figure 5 shows the theoretical contact angles calculated using Equation (2) and the measured contact angles for each steel insert. A linear curve fit was performed on the measured contact angles of the steel insert with a coefficient of determination (R^2^) of 75%. The contact angle increased as the liquid/solid surface area decreased, which fitted the observations of a typical Cassie–Baxter wetting model. It was also observed that all the measured contact angle values presented a 30°–40° increase compared with the theoretical values. Other researchers have observed this behavior on surfaces characterized by hierarchical textures. Upon wetting, the nano-scale texture further enhanced the Cassie–Baxter wetting model and the contact angle by hindering the water flow to the bottom of the nano-scale LIPSS. Additionally, the shift of the experimental data compared with the Cassie–Baxter model was linked to the change in surface properties after femtosecond laser irradiation. The topic has been widely discussed in the literature [39,40,41]. 

### 3.2. Characterization of Molded Plastic Parts

#### 3.2.1. Replication of Features

During the injection molding process, the textures (i.e., trenches, channels, and holes) were replicated as positive features on the plastic parts. These molded micro-features were analyzed to determine the replication rate of the injection molding process using two different polymers. The ratio of the average height of the features on the molded part and the average depth of the features on the steel inserts was calculated as follows:

(3) $$\mathrm{Replication}\;\mathrm{Rate}\;=\frac{\mathrm{Dimensions}\;\mathrm{of}\;\mathrm{Features}\;\mathrm{on}\;\mathrm{Polymer}\;\mathrm{Part}}{\mathrm{Dimensions}\;\mathrm{of}\;\mathrm{Features}\;\mathrm{on}\;\mathrm{Steel}\;\mathrm{Insert}}\;\times \;100\%\;$$

The replication rate provided key information about the ability of the polymer to flow into the micro-scale cavities, creating the desired micro-features on the plastic molded parts. The key geometrical properties of the molded samples using PP and PMMA are presented in Table 9 and Table 10 respectively. The aspect ratio was defined as the height of the features divided by the average width/diameter of the features on the molded parts. 

##### Replication of Feature Width

**Figure 6:**
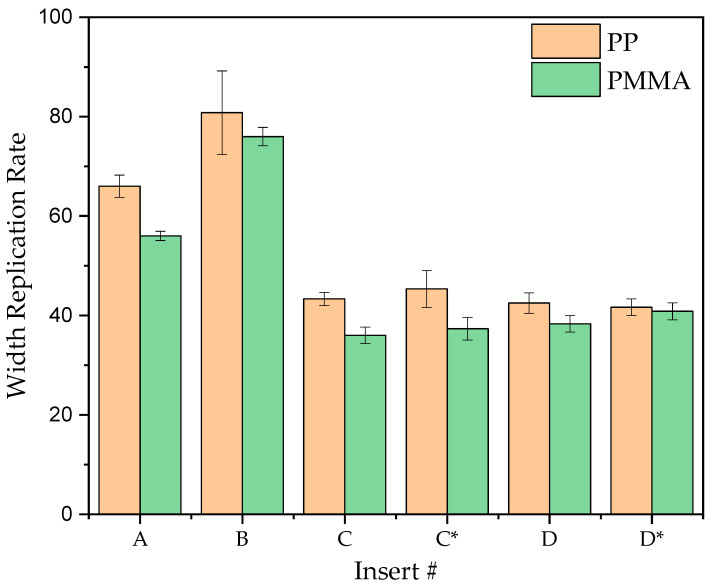
Replication rate on the feature width for PP and PMMA.

The feature widths, replicated using PP, and PMMA, are presented in Figure 6. The ratio of the average width of the features on the molded samples and the width of the features on the steel insert defined the replication rate of width. In the case of hole features (Insert B), the diameter of the cylinder on the molded samples was used as the feature width. Overall, the average width replication rate was between 42–81% for PP and 40–76% for PMMA. PP and PMMA showed similar replication rates on all inserts. regardless of the feature details or the orientation of the steel insert. Similar replication was also observed in other research works [42]. For the cylindric features, the top surface’s diameter was 20% smaller than the bottom. 

##### Replication of Feature Height

**Figure 7:**
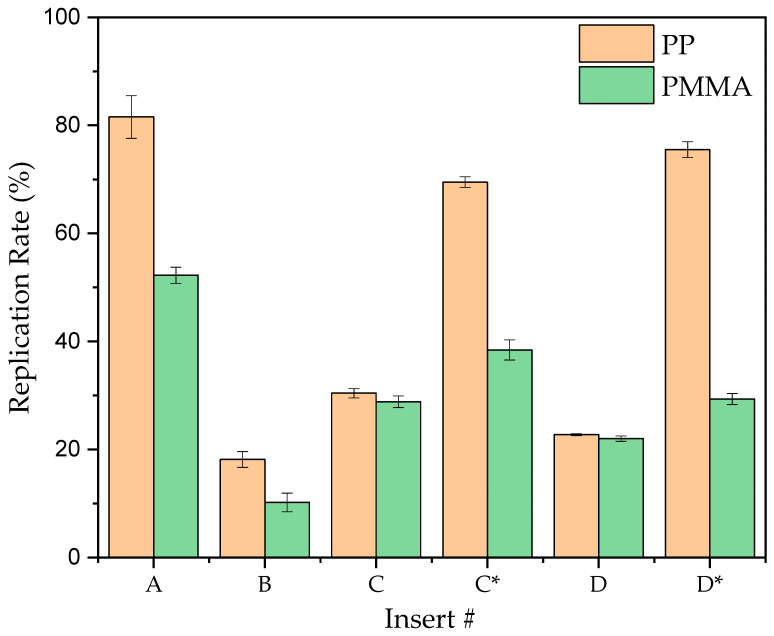
Replication rate on feature depth for PP and PMMA.

The height replication rates of the molded samples using PP and PMMA are presented in Figure 7. Overall, polypropylene samples showed a ~30% increase in replication rate for insert A, B, C*, and D* compared with PMMA. In contrast, the replication rate was identical in C and D for both materials. As the viscosity increased from 31 Pa·s for PP to 134 Pa·s for PMMA [43], the average replication rate decreased in response from 50% to 30%, respectively. A similar trend was observed on all samples fabricated with different inserts and insert orientations. Indeed, a low melt viscosity allowed for further flow into the micro-scale cavities by reducing hesitation [44]. 

The different replication rates observed with the two polymers can be associated with the thermal properties of the polymers. For amorphous materials such as PMMA, to ensure sufficient cooling and solidification, the mold temperature (80 °C) was kept below the glass transition temperature (85 °C). When the polymer melt contacted the surface of the mold at a temperature below its glass transition temperature, the intensity of the molecular movements was significantly reduced. As a result, the molecules tended to solidify before they could flow further into the micro-scale channels. For the semi-crystalline polymers, such as PP, the mold temperature was above the glass transition temperature (−20 °C for PP). The molecular chains maintained a high level of mobility when the polymer melt contacted the mold surfaces. This allowed the polymer to flow further into the micro-scale channels before the crystalline structures were formed, and the rigid part was ejected from the injection molding machine [9]. 

**Figure 8:**
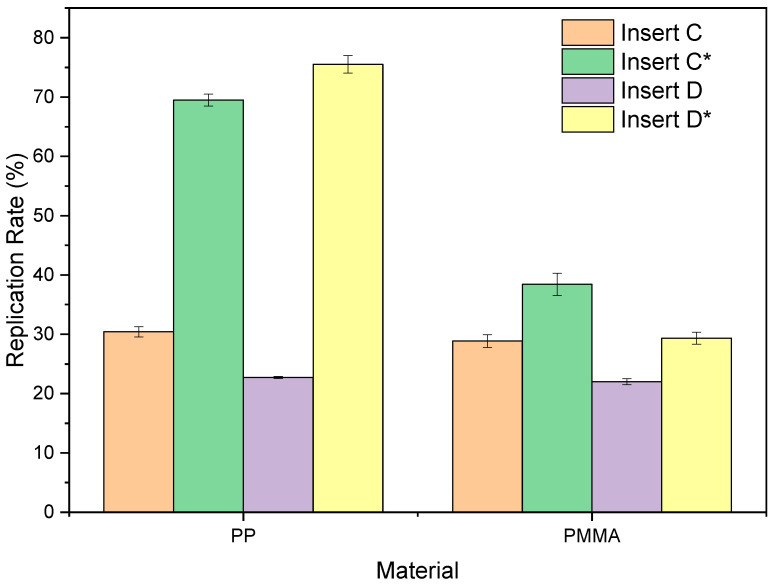
Correlation of the replication rate of depth and insert orientation.

The effect of texture orientation on polymer replication was investigated for the inserts characterized by texture directionality, namely inserts C and D. In the case of inserts C and D, the flow direction was perpendicular to the orientation of the micro-scale trenches on the inserts. In contrast, for inserts C* and D*, the polymer flow was parallel to the orientation of the micro-scale trenches. A comparison of the replication rate between C, D, and C*, D* using PP and PMMA is presented in Figure 8. It was observed that when the flow direction was perpendicular to the orientation of trenches on the steel insert (insert C, insert D), the height replication rates were at 30% and 25% for PP and PMMA, respectively. Indeed, for these texture designs, the replication was less affected by the viscosity of the polymer, but more dominated by the effect of trapped air in the micro-scale channels [24]. During the injection stage, the air trapped in the micro-cavities could not be evacuated, and its compression hindered the flow. Changing the texture orientation allowed the texture cavities to be filled more effectively, and a higher replication was observed. In the case of PP molded parts, which yielded the highest replication for the different textures, Insert C demonstrated a 39% increase when turned, while Insert D demonstrated a 53% increase. The PMMA samples showed a similar growth trend, but not as significant, due to the lack of molecular movements when molded at 80 °C. The flow was less inhibited when the parallel trench microstructures were parallel to the flow direction [45]. 

#### 3.2.2. Wetting Properties of Molded Parts

The wetting properties of the molded samples were characterized following the same approach used for the steel inserts. The wetting properties of the molded plastic are presented in Table 11. Overall, the PP parts showed higher contact angle values than the PMMA parts. PP and PMMA have different surface energy values that initially resulted in different contact angle values on the samples molded against the blank and textured steel inserts. The parts molded against blank inserts presented static contact angle values <90°, indicating that the surfaces were hydrophilic. For the samples molded against textured steel inserts, all PP samples showed static contact angle values > 90°, indicating that the surfaces were hydrophobic. For PMMA, Inserts C and D (i.e., the flow direction is perpendicular to the feature direction) showed contact angle values < 90°. Regardless of the hydrophobicity of the surfaces on the molded parts, the static contact angle increased compared with the samples molded against the blank insert for both PP and PMMA, indicating that texturing improved the hydrophobicity of the surfaces. 

To further analyze the effects of texture geometry on the static contact angle of the molded samples, only the samples fabricated using Inserts C and D are discussed in the following section. Other researchers have shown that the geometrical patterns had significant effects on the wetting behavior of the molded parts, especially when the diameter of the features above the projected surfaces was small compared with the surfaces [9,46,47]. Grewal et al. concluded that the shape of the features significantly affected the wetting behavior of water, even with a similar feature height and top-surface diameter [48]. 

In contrast with the Cassie–Baxter wetting model, the Wenzel model (Equation (4)) describes wetting behaviors where the entire surface, including the raised and indented features, is in contact with the water droplet. The liquid completely fills the cavities on the surface of a substrate and is fully in contact with the surface. In the Wenzel model, r is defined as the total contoured surface area, including the wall height of the features, divided by the apparent projected surface area of the area of interest. When r increases and the droplet is in the Wenzel state on a hydrophobic surface, the contact angle of the droplet will increase. On a hydrophilic surface, as *r* increases, the contact angle will decrease as described by the following [49]:

(4) $$\cos\left(\theta^{*}\right)=r\cos\left(\theta \right)$$

**Figure 9:**
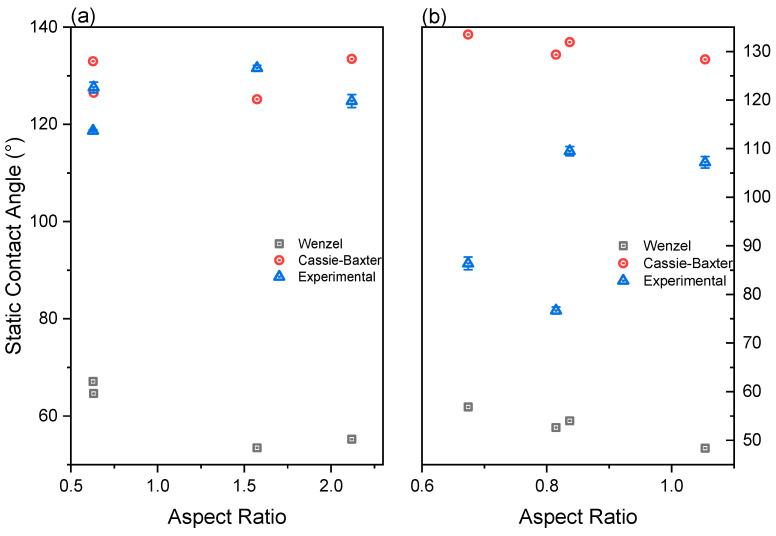
Comparison between the wetting properties of the molded part and theoretical values obtained with the Cassie–Baxter and Wenzel models: (**a**) PP and (**b**) PMMA.

Figure 9 compares the theoretical Cassie–Baxter wetting contact angle, Wenzel contact angle, and the static contact angles obtained from experiments of PP and PMMA molded parts. The static contact angles obtained from the samples molded against blank steel inserts were used as the contact angles for ideal flat surfaces when calculating the theoretical contact angle values for the Cassie–Baxter and Wenzel models using Equations (2) and (4). In the case of PP, the predicted Wenzel angles $\theta_{w}$ varied between 53°–67° depending on the aspect ratio of the surfaces, while the predicted Cassie–Baxter angles $\theta_{C-B}$ showed a hydrophobic wetting state ranging from 125°–133°. Regarding PMMA, the $\theta_{w}$ was between 48°–56°, while $\theta_{C-B}$ ranged from 128° to 133°.

In the PMMA samples, the measured static contact angles were between the CA values calculated with the models. For PMMA samples molded against Inserts C and D, where the aspect ratio of the features was less than 0.82, the surfaces displayed a hydrophilic wetting state. The contact angle decreased as the aspect ratio increased, which fitted the observation of the Wenzel wetting models. Additionally, the measured contact angle values were 25°–30° above the Wenzel Cas, but were 50°–52° lower than the Cassie–Baxter CAs. For higher aspect ratios, PMMA samples displayed hydrophobic surfaces. A significant increase was observed between 76° and 109° on the measured CAs when the aspect ratio increased from 0.82 to 0.84. Additionally, the measured CAs were closer to the Cassie–Baxter CA values at a higher aspect ratio and did not decrease when the aspect ratio increased as in the Wenzel condition. 

As the measured CAs were between the Wenzel and Cassie–Baxter calculated CAs, it can be hypothesized that the textured surfaces produced on PMMA followed neither the Wenzel wetting state nor the Cassie–Baxter state, but an intermediate composite state. According to other research [50,51], pure Wenzel and Cassie–Baxter wetting states rarely occur. The intermediate state, which is called composite or mixed wetting state, was observed by many other researchers [4,52,53]. In this state, the water droplet partially penetrates the valleys and comes in contact with the rough solid surfaces, while part of the water droplet sits on the air pocket between the bottom of the liquid surface and the base of the solid features. According to Whyman et al., the wetting of inherently hydrophilic surfaces is always energetically favorable, while Cassie–Baxter wetting is possible only when air is trapped in the rough surfaces [54]. He et al. observed the measured contact angle of 152.5° on a rough polydimethylsiloxane (PDMS) surface patterned with perfect square pillar arrays, which lies between the theoretically calculated Cassie–Baxter (165.7°) and the Wenzel angles (121°) [52]. The reason for this complex wetting state is still an open question in the literature. Lamufa et al. reported a mixed state with an average static contact angle of ~110°, close to the observations reported in this research [55]. However, recent studies have reported that this composite wetting also exists on complicated hierarchical surfaces, such as rose petals [56]. 

In the case of PP parts, the measured static contact angles were close to the Cassie–Baxter angles. The differences between the measured CAs and $\theta_{C-B}$ were <10° in all cases. Even for low aspect ratios (AR < 0.8), the measured CAs were >90°, indicating hydrophobic surfaces. As the aspect ratio increased >1.5, more hydrophobic surfaces were observed as the measured CAs increased. The different wetting behavior of PP and PMMA at low aspect ratio ranges was reconducted to the different surface energy for the two polymers, which created different adhesion behavior at the liquid–solid surfaces. The surface energy of PMMA averages 40.3 ± 0.3 N/m, while the surface energy of PP is lower at 31.5 ± 0.5 N/m [57,58]. Lower surface energy materials exhibit higher static contact angle values on naturally rough surfaces than materials with a high surface energy [59]. 

As the laser-induced surface morphology is typically complex and usually lacks homogeneity due to the Gaussian profile of laser beams, it is difficult to directly describe the microscopic wetting state on the steel insert and molded samples. Therefore, further modeling work will be required to understand the wetting phenomenon better.

## 4. Conclusions

This work assessed the effect of different textures on parts molded using different polymers. From the experimental results, the following conclusions can be drawn:

- The replication rate correlated with the polymer’s melt viscosity and thermal properties. A lower-viscosity polymer resulted in a higher degree of replication. Keeping the mold temperature above the glass transition temperature for semi-crystalline polymers resulted in a higher degree of replication.
- The replication depended on the orientation of inserts for those with a directional geometry. Altering the texture orientation produced differences in the replicated texture. Texture geometries that promoted air entrapment were characterized by a higher hesitation and lower replication.
- Hierarchical texturing created a hydrophobic wetting behavior. On the steel inserts, the experimental static contact angles showed a 30°–40° increase compared with the Cassie–Baxter contact angle values when only the geometrical properties of the micro-features were considered.
- The wetting behavior of the molded polymer samples showed different wetting states due to differences in the surface energy. PP samples exhibited a Cassie–Baxter wetting state, while the PMMA samples showed a composite wetting state of Cassie–Baxter and Wenzel states. For the lower aspect ratio textures, the wetting behavior followed the Wenzel model, while the Cassie–Baxter wetting model was predominant with a higher aspect ratio. The hierarchical structures increased the contact angles overall, but the nano-scale features’ effects need further investigation.

## Figures and Tables

**Table 1:** Polymers used in micro-injection molding.

Polymer	Abbreviation	Applications
Polyoxymethylene	POM	Micro filters and gears
Liquid Crystal Polymers	LCP	Micro connectors, micro-electronic devices
Polycarbonate	PC	Optical lenses, sensor discs
Polymethylmethacrylate	PMMA	Optical lenses, optical fiber connectors
Polypropylene	PP	Self-cleaning surfaces, packaging
Polylactic acid	PLA	Biomedical implants

**Table 2:** Common texturing technologies used for injection molding applications.

Technology	Feature Dimensions	Texturing Speed	Cost	Advantage	Limitation	Reference
Electroplating	20–50 µm	Low	Low	Component is resistant to tarnishing	Difficult to dispose of chemical waste	Genolet et al. [24]
Anodization	>0.1 µm	Low	Low	Control of surface finish	Can only be applied to aluminum	Santos et al. [25]
Chemical Etching	Min. dimension depends on the masking used	Low	Low	Large area texturing	Undercut Difficult to control	Bruzzone et al. [26]
Lithography	Min. 5 nm	Low	High	Very precise structures	Expensive Time-consuming	Unno et al. [27]
LIGA	Min. 500 nm	Low	High	Very precise structures	Expensive Time-consuming No Drafts	Saile et al. [28]
Laser Writing	Min. 20 µm	Low	Med	3D shape texturing Relatively inexpensive tooling	Heat affect zone (HAZ). Presence of a recast layer	Gregorčič et al. [29]
Ultrafast laser texturing	Max 100 µm Min. 100 nm	High	Med	Hierarchical structures with up to 2 levels	Regularity of pattern, shape of the structures challenging	Piccolo et al. [8]

**Table 3:** Laser system parameters.

Parameter	Unit	Value
Max power	W	3 (IR)–1.8 (GR)
Max pulse energy	µJ	30 (IR)–18 (GR)
Oscillator frequency	MHz	40
Pulse repetition rate	kHz	1000 (IR & GR)
M^2^	-	1.3

**Table 4:** SEM images of the textured surfaces on steel inserts.

Insert Index	Low Magnification	High Magnification
A	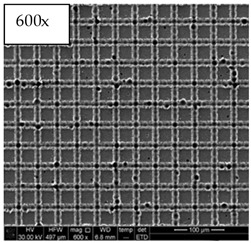	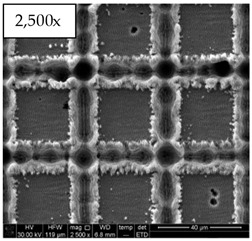
B	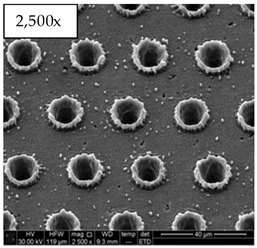	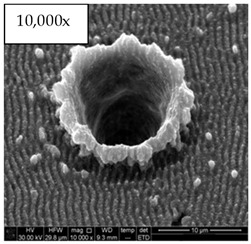
C	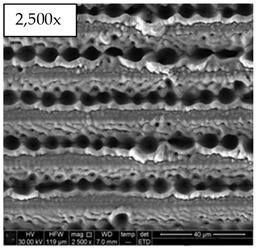	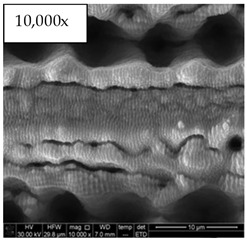
D	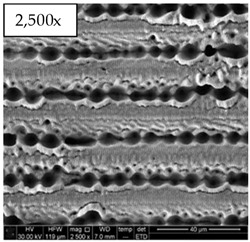	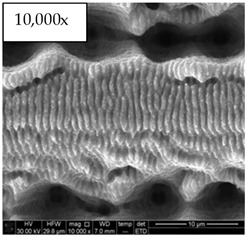

**Table 5:** Material properties of the selected polymers.

Material	Glass Transition Temperature (°C)	Melt Flow Index @230 °C/5.0kg (g/10 min)	Density (kg/m^3^)	Melting/Softening Temperature (°C)
PMMA	85	24	1180	180–200
PP	−20	6	905	180–220

**Table 6:** Processing parameters of the micro-injection molding process.

Processing Parameters	Material	
	PP	PMMA
Shot Size (mm)	10	10
Nozzle Temperature (°C)	230	230
Injection Velocity (mm/s)	158	158
Packing Pressure (MPa)	130	130
Packing Time (s)	3	3
Mold Temperature (°C)	80	80
Cooling Time (s)	30	25

**Table 7:** Dimensional characteristics of the steel inserts.

	Width/Diameter (μm)		Depth (μm)		AR	TSA (μm^2^)
Insert	Avg.	Std. Dev.	Avg.	Std. Dev.		
Insert A	20.0	0.35	5.4	1.96	0.2	10,240
Insert B	12.5	0.49	10.9	2.69	0.9	18,082
Insert C	15.0	0.25	15.4	1.26	1.0	11,025
Insert D	12.0	0.30	14.1	1.80	1.2	15,167

**Table 8:** Wetting properties of the steel inserts.

	Static Contact Angle (°)		Percent Change (%)
Insert ID	Avg.	Std. Dev.	
Blank	74.6	1.1	N/A
Insert A	145.0	2.8	94.4
Insert B	126.7	3.5	69.8
Insert C	135.4	2.5	81.5
Insert D	135.0	2.9	81.0

**Table 9:** Wetting properties of steel inserts compared with the Cassie–Baxter model.

Insert ID	Width (µm)		Height (µm)	
	Average	Std. Dev.	Average	Std. Dev.
Blank	N/A	N/A	N/A	N/A
Insert A	13.2	0.9	4.4	0.49
Insert B	10.1	2.1	2.0	0.31
Insert C	6.5	0.4	4.1	0.26
Insert C*	6.8	1.1	10.7	0.31
Insert D	5.1	0.5	3.2	0.05
Insert D*	5	0.4	10.6	0.41

**Table 10:** Replication quality parameters for parts molded with poly(methyl methacrylate).

Insert ID	Width (µm)		Height (µm)	
	Average	Std. Dev.	Average	Std. Dev.
Blank	N/A	N/A	N/A	N/A
Insert A	11.2	0.4	2.8	0.16
Insert B	9.5	0.4	1.1	0.37
Insert C	5.4	0.5	4.4	0.32
Insert C*	5.6	0.7	5.9	0.57
Insert D	4.6	0.4	3.1	0.14
Insert D*	4.9	0.4	4.1	0.29

**Table 11:** Wetting properties of molded PP and PMMA parts.

Material	PP		PMMA	
Static Contact Angle (°)	Average	Std. Dev.	Average	Std. Dev.
Blank	75.7	0.5	69.2	0.6
Insert A	139	2.1	97	0.5
Insert B	116.9	4.1	96.6	1.6
Insert C	127.6	2.2	76.7	1.4
Insert C*	131.6	1.1	107.2	2.4
Insert D	118.7	0.3	86.4	2.6
Insert D*	124.8	2.7	109.5	1.9

## Data Availability

Not applicable.
